# Supplementary material for: Variation and internal-external driving forces of grey water footprint efficiency in China’s Yellow River Basin
Source: PLoS One. 2023 Mar 22;18(3):e0283199. doi: 10.1371/journal.pone.0283199 (PMC10032503; doi:10.1371/journal.pone.0283199)
Supplement: Attachment — Submitted filename: Response to Reviewers.docx [file pone.0283199.s003.docx]

**We thank the Editor and Reviewers for his/her constructive and helpful suggestions and have now incorporated all of them into the revised version of the paper. Below are the details of our responses.**

**Reviewer #1:**

*This manuscript investigated the spatial and temporal variation of the grey water footprint (GWF) efficiency based on the measurement of the GWF efficiency, the kernel density estimation and the Dagum Gini coefficient method. The paper is written in sound English, but with some grammar errors. Detailed comments are as follows:*

*1. “For demonstrating this assessment approach, China's Yellow River Basin is chosen as an example.”, spatial and temporal scales should be clarified for the empirical study in this sentence.*

**Our responses:** We are very grateful to the reviewer for this suggestion. We have modified this sentence in the abstract to clarify the spatial and temporal scale of the study. The sentence is presented in a new expression——"For demonstrating this assessment approach, nine provinces in China's Yellow River Basin from 2005 to 2020 are chosen for the study".

*2. “Inter-regional variation is the primary source of spatial variation, with an average contribution of 73.39%.”, please clarify inter-regional variation of what?*

**Our responses:** We are very grateful to the reviewer for this suggestion. This sentence in the abstract has been modified to "Inter-regional variation of the GWF efficiency is the primary source of spatial variation, with an average contribution of 73.39%".

*3. A map should be added to provide a detailed description of the study area.*

**Our responses:** We thank the reviewer for the suggestion and in the revised version we have added a map to provide a detailed description of the study area (see pages 6-7). The detail additions are as follows:

# Overview of the study area

The Yellow River Basin in China spans nine provinces, connecting the Qinghai-Tibet Plateau, the Loess Plateau and the North China Plain, making it an important ecological and economic zone. With only 27% of China's average per capita water resources, the Yellow River Basin is responsible for supplying water to 12% of the country's population, 17% of its arable land and more than 50 large and medium-sized cities. In addition, the Yellow River Basin is an important industrial production area in China, with abundant traditional resources such as coal, oil, natural gas and non-ferrous metals. However, the rough development has led to excessive consumption of resources and severe water pollution problems in the river basin. In 2022, the report of the 20th National Congress of the Communist Party of China (CPC) put forward "promoting the ecological protection and high-quality development of the Yellow River basin" as a major strategy to facilitate coordinated regional development. Although the Yellow River has been managed to a certain extent, the problem of fragile water ecology in the basin is still prominent. Coordinating the relationship between water pollution and economic development is a must for the implementation of high-quality development in the Yellow River Basin.

Therefore, nine provinces in China's Yellow River Basin, including Qinghai, Sichuan, Gansu, Ningxia, Inner Mongolia, Shanxi, Shaanxi, Henan and Shandong, are taken as the research objects. The GWF and its efficiency are measured for the nine provinces between 2005 and 2020. Fig 1 shows an overview of the study area.

**Fig 1. Overview of the study area**

*4. As to the numbers in the text and tables, I suggest the authors keep only two decimal places in a number.*

**Our responses:** We are grateful to the reviewer for the suggestion and we have modified the relevant numbers to keep only two decimal places in the new revised version.

*5. In methods and data, the use of operator signs in formulas should be uniform.*

**Our responses:** We thank the reviewer for their careful comment. We have uniformed the operator signs in the formulas. See formulas (1), (12) and (21) on pages 7, 10 and 12, respectively.

*6. Natural and social conditions differ significantly within provinces, such as Inner Mongolia. Is the provincial-scale analysis too general? If possible, analysis at a more precise scale could be more convincing.*

**Our responses:** We are grateful to the reviewer for the suggestion. Considering the availability of data, the nine provinces in the Yellow River Basin are chosen as the main research objects in this paper. Study at a more micro-scale, such as cities or counties, is a further direction for expansion. In order to carry out the analysis on a more precise scale, we have revised the description of the variation characteristics of the GWF and its efficiency in different regions (see pages 16-17). The detailed changes are as follows:

To observe the changes in GWF efficiency of different provinces, Fig 3 reports the GWF efficiency and the proportion of the GWF of different sectors at four-time points: 2005, 2010, 2015 and 2020. As can be seen, the GWF efficiency varies considerably between provinces, showing a spatial distribution pattern of "low in the western and high in the eastern". The GWF efficiency of Shandong province in the downstream remains the highest, while Qinghai province in the upstream is the lowest. Qinghai is the source of the Yellow River and has a relatively good water environment, with a low GWF of 6.82 billion m^3^, which is around 2/5 of the average GWF of the basin as a whole. However, as an economically underdeveloped region, Qinghai is still in the early stages of industrial development, and this situation has not been effectively improved over the years. The average GDP of Qinghai is only 186.14 billion yuan, much lower than the overall basin average GDP of 1733.16 billion yuan, which causes the GWF efficiency to be small. This result is also reflected in the study by Han et al. [10]. Differently, the other upstream provinces (Sichuan, Gansu and Ningxia) have received a faster increase in GWF efficiency, due to a slight decrease in grey water emissions and a rapid rise in GDP. In comparison, Shandong province in the downstream has secured an increase in GWF efficiency from 46.82 yuan/m^3^ to 294.63 yuan/m^3^ in parallel with stable socio-economic development. The reason is that Shandong, as an estuary province of the Yellow River, has a comparative advantage and is better developed in terms of economy, industry and technology [3]. Especially, core cities in Shandong, such as Jinan, have made efforts to promote high-quality development, with the growth rate of major economic indicators remaining in the top echelon of the province and strategic emerging industries showing strong and resilient development trends.

In addition, the downstream province of Henan also experiences significant growth in GWF efficiency during the observation period, with an 8.24-fold increase. As a central agricultural province in the Yellow River Basin, Henan has accelerated its agricultural emission reduction efforts in recent years, and has been effective in controlling greywater emissions, decreasing from 45.01 billion m^3^ to 28.38 billion m^3^. Similarly, the GWF efficiency of Shanxi and Shaanxi in the midstream has risen rapidly, with increases of 230.85 yuan/m^3^ and 206.64 yuan/m^3^, respectively. However, Inner Mongolia, another midstream province, has seen relatively little improvement in the GWF efficiency. Despite its economic growth over the years, there has been no significant reduction in grey water emissions. Inner Mongolia has a sizeable east-west span, with a highly uneven distribution of resources and economic development. The western economic zone of Inner Mongolia, centered on Hohhot, Ordos and Baotou, is the main source of the province's economy, while the other regions have experienced very slow economic growth. Moreover, the industry based on energy sources such as coal has led to serious water pollution.

*7. According to Fig. 1, both TGWF and GWF efficiency were mutated in 2020. An analysis of this phenomenon can be added to the text.*

**Our responses:** Thanks to the reviewer's suggestion, we have added an analysis of the phenomenon of mutation in the GWF and its efficiency in 2020 (see page 15). The detail additions are as follows:

In addition, both the GWF and its efficiency change abruptly in 2020. Compared to 2019, the domestic GWF is significantly higher in 2020, leading to an increase in the total GWF. However, due to macro factors such as the New Crown epidemic, the growth in GDP of 2020 is slight, which results in a significant decrease in the GWF efficiency. It indicates that urban wastewater treatment in the basin needs to be strengthened. In response, in 2021, China's Development and Reform Commission and Ministry of Housing and Construction jointly issue the Implementation Plan for the Urban Sewage and Waste Treatment in the Yellow River Basin during the "14th Five-Year Plan" period to improve the domestic sewage treatment rate.

*8. The graphic style of Fig. 3 does not show well the three-dimensional distribution of information. Try adjusting the angle or changing the layout to make the information clear.*

**Our responses:** We thank the reviewer for the suggestion, and in the new revised version, we have replotted the kernel density estimates of GWF efficiency with 2005, 2008, 2011, 2014, 2017 and 2020 as typical years to make the information clear (see page 19).

*9. Some grammar problems can be checked.*

**Our responses:** The article has been checked in detail and we have improved the text for possible grammar problems.
